# Assessing the Relationship Between Steatotic Liver Disease and Barrett’s Esophagus: A Cross-Sectional Analysis of 5507 Screening Participants

**DOI:** 10.1055/a-2763-8210

**Published:** 2026-03-20

**Authors:** Nikolaus Götz, Andreas Völkerer, Hannah Hofer, Sarah Wernly, Franz Singhartinger, Ewald Wöll, Elmar Aigner, Maria Flamm, Christian Datz, Bernhard Wernly

**Affiliations:** 1 31507 Paracelsus Medical UniversitySalzburgSalzburgAustria

**Keywords:** Liver disease, Esophageal neoplasia, Public Health, Barrett Ösophagus, Lebererkrankung, Ösophageale Neoplasie, Öffentliche Gesundheit

## Abstract

**Background:**

Barrett’s esophagus (BE) is the main precursor of esophageal adenocarcinoma (~2% prevalence in Western adults). Steatotic liver disease (SLD), affecting ~25% of adults, is linked to systemic inflammation, but its relationship with BE is unclear.

**Methods:**

In the population-based Salzburg Colon Cancer Prevention Initiative (2007–2020), we analyzed 5507 asymptomatic screening participants. SLD was diagnosed by ultrasound and classified as MASLD or MetALD; BE was confirmed endoscopically and histologically. Associations were assessed using multivariable logistic regression adjusting for demographic, metabolic, and clinical factors.

**Results:**

SLD was present in 2550 participants and clustered with diabetes and metabolic syndrome. BE was found in 62 individuals (1.0% without SLD, 1.5% with MASLD, 1.1% with MetALD; P = 0.28). After adjustment, MASLD (OR 1.28; 95% CI 0.74–2.21) and MetALD (OR 0.66; 95% CI 0.16–2.85) were not significantly associated with BE. Female sex was protective (OR 0.26; 95% CI 0.13–0.50), while hiatal hernia increased BE risk (OR 2.05; 95% CI 1.16–3.60).

**Conclusion:**

In this population-based cohort, MASLD and MetALD were not clearly associated with BE. Current data do not support incorporating SLD status into BE screening algorithms, although modest risk increases cannot be ruled out.

## Zusammenfassung

**Hintergrund:**

Der Barrett-Ösophagus (BE) ist die zentrale Vorstufe des Ösophagusadenokarzinoms (~2% in westlichen Populationen). Steatotic Liver Disease (SLD; ~25% Prävalenz) ist mit systemischer Inflammation verknüpft; ein Zusammenhang mit BE ist unklar.

**Methoden:**

In der bevölkerungsbasierten Salzburg Colon Cancer Prevention Initiative (2007–2020) wurden 5507 asymptomatische Personen untersucht. SLD wurde sonografisch erfasst und als MASLD bzw. MetALD eingeteilt; BE endoskopisch und histologisch gesichert. Assoziationen wurden mittels multivariabler logistischer Regression (Anpassung an demografische, metabolische und klinische Faktoren) analysiert.

**Ergebnisse:**

SLD lag bei 2550 Teilnehmenden vor und war mit Diabetes und metabolischem Syndrom vergesellschaftet. BE fand sich bei 62 Personen (1,0% ohne SLD, 1,5% bei MASLD, 1,1% bei MetALD; P = 0,28). Weder MASLD (OR 1,28; 95%-KI 0,74–2,21) noch MetALD (OR 0,66; 95%-KI 0,16–2,85) zeigten eine signifikante Assoziation mit BE. Weibliches Geschlecht war protektiv (OR 0,26), eine Hiatushernie mit erhöhtem Risiko verbunden (OR 2,05).

**Schlussfolgerung:**

In dieser Kohorte bestand kein eindeutiger Zusammenhang zwischen MASLD/MetALD und BE. Der SLD-Status sollte derzeit nicht routinemäßig in BE-Screeningstrategien einfließen; geringe Risikoerhöhungen können jedoch nicht ausgeschlossen werden.

## Introduction

BE, characterized by the substitution of the normal distal esophageal squamous lining with intestinal-type columnar epithelium containing goblet cells, represents the primary precursor lesion for esophageal adenocarcinoma. According to recent meta-analyses, this condition affects approximately 1.5% of the general population, with an estimated annual malignant transformation rate to esophageal carcinoma (EAC) of 0.2% to 0.3% 1 2 3 . The increasing rates of EAC, combined with its poor outcomes due to late diagnosis, highlight the critical need to identify risk factors for BE and its advancement 4 5 6 7 .

Advanced age, male sex, gastroesophageal reflux disease, obesity, and tobacco use are well-established risk factors, yet these variables alone do not account for the continuing rise in adenocarcinoma incidence observed in Western populations over the past three decades 8 9 10 11 12 .

SLD – now classified into MASLD and MetALD – affects approximately one quarter of adults in industrialized countries and clusters with the metabolic-syndrome traits that underlie several gastrointestinal malignancies 13 14 .

Hepatic steatosis is associated with systemic low-grade inflammation, altered adipokine signaling, and insulin resistance – biological mechanisms that may plausibly contribute to metaplastic transformation within the esophageal epithelium 15 16 17 18 .

However, current evidence directly linking SLD to the development of BE remains sparse and inconclusive. Existing case-control studies have been limited by small sample sizes, inconsistent diagnostic criteria for hepatic steatosis, and inadequate adjustment for potential metabolic confounders 19 20 21 22 .

We therefore conducted a large, population-based endoscopy-screening study to quantify the association between MASLD and MetALD and the prevalence of BE. We additionally examined whether age, sex, and metabolic traits modified this relationship and whether noninvasive markers of hepatic fibrosis were associated with Barrett’s risk among participants without clinically defined SLD.

## Methods

### Study Population

This investigation draws upon data from Sakkopi, a longitudinal screening cohort designed to detect colorectal neoplasia, conducted at a single academic center in Austria between January 2007 and March 2020. The study protocol was approved by the local ethics committee (approval number 415-E/1262), and all participants provided written informed consent in accordance with the Declaration of Helsinki.

Of 5516 asymptomatic individuals who underwent comprehensive screening evaluation, 5507 participants with complete datasets enabling classification of SLD and evaluation of BE were included in the present analysis. Upper endoscopy was performed opportunistically in conjunction with colonoscopy screening to maximize detection of upper gastrointestinal pathology at minimal additional patient burden. Asymptomatic men and women aged 45–75 years were invited to participate in the Sakkopi, irrespective of sex; thus, screening eligibility and age at entry did not differ between males and females.

All participants were asymptomatic volunteers without specific upper gastrointestinal complaints, alarm symptoms, or indication for surveillance endoscopy. Exclusion criteria included prior diagnosis of esophageal cancer, previous esophageal surgery, active malignancy, or incomplete endoscopic or ultrasound examinations. Data acquisition encompassed endoscopic observations, comprehensive clinical histories, anthropometric indices, lifestyle-related factors, biochemical profiles, and findings from abdominal ultrasonography.

### SLD – Classification

SLD was identified using standardized abdominal ultrasonography at the baseline screening examination. All scans were performed by trained personnel following a uniform protocol. Where available, controlled attenuation parameter (CAP) measurements obtained by transient elastography were used to corroborate the sonographic impression of fatty infiltration, thereby increasing diagnostic robustness. The subsequent categorization of SLD adhered to the 2023 consensus nomenclature and incorporated both metabolic profile and alcohol intake patterns.

MASLD was defined by evidence of hepatic fat accumulation together with at least one cardiometabolic abnormality, including overweight or obesity, type 2 diabetes mellitus, arterial hypertension, dyslipidemia, or dysglycemia. In addition, alcohol intake had to remain below 30 g/day for men and below 20 g/day for women.

MetALD encompassed individuals fulfilling MASLD criteria who, in contrast, reported moderate alcohol exposure quantified as 30–60 g/day for men and 20–50 g/day for women.

Alcohol-associated liver disease (ALD) referred to cases with hepatic lipid deposition in the context of substantial alcohol use, defined as ≥60 g/day for men or ≥50 g/day for women, in the absence of cardiometabolic disturbances sufficient to meet MASLD requirements.

Cryptogenic steatotic liver disease captured participants with liver fat on imaging who did not exhibit cardiometabolic risk factors and did not report alcohol intake above MetALD/ALD thresholds (≥30 g/day in men or ≥20 g/day in women), and therefore were considered to have steatosis of unexplained origin.

Participants without ultrasonographic signs of hepatic fat accumulation were categorized as having no SLD and served as the reference group in all comparative analyses.

### BE – Assessment

BE diagnosis required both endoscopic visualization of columnar-lined esophagus extending at least 1 cm proximal to the gastroesophageal junction and histological confirmation of specialized intestinal metaplasia with goblet cells on targeted biopsies. Endoscopy was performed by experienced gastroenterologists using high-definition white-light endoscopy. Cases with endoscopic suspicion but lacking histological verification of intestinal metaplasia were classified as negative for BE. Histological specimens were further categorized to identify the presence and grade of dysplastic changes according to established criteria (non-dysplastic Barrett, low-grade dysplasia, high-grade dysplasia). The presence of hiatal hernia was recorded during endoscopy and defined as displacement of the gastroesophageal junction ≥2 cm proximal to the diaphragmatic hiatus.

### Metabolic Syndrome and Fibrosis Assessment

Metabolic syndrome was defined according to Adult Treatment Panel III (ATP III) criteria, requiring the presence of at least three of the following five components: waist circumference >102 cm in men or >88 cm in women; triglycerides ≥150 mg/dL or lipid-lowering therapy; HDL cholesterol <40 mg/dL in men or <50 mg/dL in women; blood pressure ≥130/85 mmHg or antihypertensive treatment; and fasting glucose ≥100 mg/dL or treatment for diabetes.

Non-invasive liver fibrosis/steatosis assessment was performed using established scoring systems including the Fibrosis-4 (FIB-4) index, AST-to-platelet ratio index (APRI), and fatty liver index (FLI). The FIB-4 index was calculated as: age (years) × AST (U/L)/[platelet count (10⁹/L) × √ALT (U/L)]. The APRI score was calculated as: [AST (U/L)/upper limit of normal] × 100/platelet count (10⁹/L). The FLI was calculated using the validated formula incorporating Body mass index (BMI), waist circumference, triglycerides, and GGT.

### Covariates and Data Collection

Demographic variables included age, sex, and educational attainment, which was stratified according to a modified International Standard Classification of Education framework into three categories: lower education (primary schooling), intermediate education (secondary education and vocational qualifications), and higher education (tertiary academic achievement or equivalent professional training). Anthropometric measurements including height, weight, and waist circumference were obtained using standardized protocols. BMI was calculated as weight in kilograms divided by height in meters squared.

Lifestyle factors were assessed through structured questionnaires. Alcohol consumption was quantified in gram per day. Smoking history was classified as never-smoker, former smoker, or current smoker and subsequently dichotomized as ever-smoker versus never-smoker for regression analyses. Medication use, including proton pump inhibitors, was recorded based on self-report and verified against prescription records where available.

Fasting venous blood samples were collected after an overnight fast of at least 8 hours. Laboratory analyses were performed using standardized automated assays at the institutional clinical laboratory, including measurement of liver enzymes (AST, ALT, GGT, alkaline phosphatase), lipid panel (total cholesterol, HDL cholesterol, LDL cholesterol, triglycerides), glucose metabolism markers (fasting glucose, HbA _1c_ ), and inflammatory markers (C-reactive protein). An oral glucose tolerance test was performed in participants without known diabetes.

### Statistical Analysis

Descriptive statistics are presented as medians with interquartile ranges (IQR) for continuous variables due to non-normal distributions, and as frequencies with percentages for categorical variables. Between-group comparisons employed the Kruskal-Wallis test for continuous variables and Pearson’s chi-square test for categorical variables.

The primary analytical approach utilized multivariable logistic regression to examine the association between SLD subtypes and BE prevalence, with participants without SLD serving as the reference category. Sequential modeling strategies included: (1) unadjusted analysis; (2) adjustment for demographic factors (age, sex); and (3) full adjustment for age, sex, metabolic syndrome, proton pump inhibitor use, hiatal hernia, and smoking status.

Interaction terms were systematically tested to evaluate potential effect modification by age (continuous), sex, metabolic syndrome, proton pump inhibitor use, hiatal hernia, and smoking status. Interactions were assessed by including multiplicative terms in logistic regression models, with statistical significance determined by the Wald test for the interaction term coefficient.

Secondary analyses examined the association between non-invasive fibrosis / steatosis markers (FIB-4, APRI, FLI, CAP, liver stiffness) and BE risk specifically among participants without clinically defined SLD, using simple logistic regression with continuous predictor variables.

Given the low Barrettʼs prevalence (1.1%), the study had limited power to detect small effect sizes (OR <2.0), but adequate power for clinically meaningful associations. Results are presented as odds ratios (OR) with corresponding 95% confidence intervals (CI), with statistical significance defined as two-sided p<0.05. All computational analyses were performed using Stata statistical software version 17 (StataCorp, College Station, TX, USA).

## Results

### Baseline Characteristics

Among 5507 participants included in the analysis, 2957 (54%) had no SLD, 2027 (37%) were classified as MASLD, 223 (4%) as MetALD, 79 (1%) as ALD, and 221 (4%) as cryptogenic SLD. Baseline characteristics stratified by SLD status are presented in Table 1 .

**Table:** **Table 1** Baseline demographic, clinical, metabolic, and endoscopic characteristics of the study population stratified by SLD – status (no SLD, MASLD, MetALD, ALD). Continuous variables are presented as median (interquartile range), and categorical variables as number (percentage). P-values refer to overall group differences and were calculated using Kruskal–Wallis tests for continuous variables and χ² tests for categorical variables.

Characteristic	Total (N=5507)	No SLD (N=2957)	MASLD (N=2027)	MetALD (N=223)	ALD (N=79)	Cryptogenic SLD (N=221)	p-value
**Demographics**							
Age (years)	58 (52–66)	56 (51–65)	59 (53–66)	62 (55–68)	59 (52–66)	63 (54–70)	<0.001
Male sex, n (%)	2861 (52)	1270 (43)	1270 (63)	163 (73)	70 (89)	88 (40)	<0.001
**Anthropometric Data**							
Body mass index (kg/m²)	27 (24–30)	25 (23–27)	29 (26–32)	28 (26–30)	28 (27–31)	29 (25–33)	<0.001
Waist circumference (cm)	96 (88–105)	91 (83–98)	103 (97–111)	102 (95–109)	106 (98–112)	102 (93–113)	<0.001
**Laboratory Values**							
AST (U/L)	21 (17–26)	19 (17–23)	22 (18–28)	24 (19–31)	27 (20–36)	21 (17–27)	<0.001
ALT (U/L)	21 (15–30)	18 (14–23)	26 (19–38)	28 (20–40)	31 (22–44)	23 (17–33)	<0.001
GGT (U/L)	26 (17–44)	21 (15–32)	33 (22–54)	43 (28–69)	70 (45–129)	28 (20–47)	<0.001
HDL cholesterol (mg/dL)	56 (47–67)	61 (51–72)	50 (42–60)	52 (45–63)	54 (47–67)	50 (42–58)	<0.001
Fasting glucose (mg/dL)	97 (90–106)	94 (88–101)	102 (94–113)	101 (95–116)	103 (98–112)	100 (94–117)	<0.001
HbA _1c_ (%)	5.5 (5.3–5.8)	5.4 (5.2–5.7)	5.6 (5.3–5.9)	5.6 (5.3–5.9)	5.5 (5.2–5.8)	5.7 (5.4–6.0)	<0.001
C-reactive protein (mg/dL)	0.2 (0.1–0.4)	0.1 (0.1–0.3)	0.2 (0.1–0.5)	0.2 (0.1–0.4)	0.2 (0.1–0.5)	0.3 (0.2–0.5)	<0.001
**Metabolic Syndrome**							
Metabolic syndrome, n (%)	2258 (41)	706 (24)	1226 (60)	128 (57)	54 (68)	144 (65)	<0.001
Diabetes mellitus, n (%)	798 (14)	187 (6)	477 (24)	57 (26)	16 (20)	61 (28)	<0.001
Arterial hypertension, n (%)	3798 (69)	1779 (60)	1606 (79)	177 (79)	72 (91)	164 (74)	<0.001
**Lifestyle Factors**							
Alcohol consumption (EASL categories) (%/n)							<0.001
<2o/30 gram/day (F/M)	89% (4,613)	91% (2,560)	100% (2027)	0% (0)	0% (0)	96% (26)	
20–50/30–60 gram/day (F/M)	9% (443)	8% (219)	0% (0)	100% (223)	0% (0)	4% (1)	
>50/60 gram/day (F/M)	2% (103)	1% (24)	0% (0)	0% (0)	100% (79)	0% (0)	
PPI use, n (%)	400 (7)	163 (6)	187 (9)	20 (9)	7 (9)	23 (10)	<0.001
**Endoscopic Findings**							
Hiatal hernia, n (%)	2945 (53)	1519 (51)	1132 (56)	123 (55)	51 (65)	120 (54)	0.007
**Fibrosis Markers**							
FIB-4 score	1.14 (0.89–1.50)	1.12 (0.89–1.47)	1.16 (0.90–1.51)	1.25 (0.98–1.66)	1.33 (1.02–1.95)	1.13 (0.83–1.52)	<0.001
Fatty liver index	50 (22–78)	28 (13–52)	75 (53–89)	74 (48–89)	86 (70–93)	75 (44–89)	<0.001
Liver stiffness (kPa)	4.6 (3.7–5.8)	4.3 (3.5–5.3)	4.9 (3.9–6.2)	4.9 (4.1–6.9)	5.8 (5.2–7.4)	4.6 (3.7–5.6)	<0.001
**Primary Outcome**							
Barrettʼs esophagus, n (%)	62 (1.1)	29 (1.0)	30 (1.5)	2 (0.9)	1 (1.3)	0 (0)	0.24
Non-dysplastic	59 (1.1)	27 (0.9)	29 (1.4)	2 (0.9)	1 (1.3)	0 (0)	
Low-grade dysplasia	2 (0.04)	1 (0.03)	1 (0.05)	0 (0)	0 (0)	0 (0)	
High-grade dysplasia	1 (0.02)	1 (0.03)	0 (0)	0 (0)	0 (0)	0 (0)	

Participants with any form of SLD were significantly older than those without SLD, with median ages ranging from 59 years (MASLD) to 63 years (cryptogenic SLD) compared to 56 years in those without SLD (p<0.001). Male sex predominated in metabolic and alcohol-related SLD groups (MASLD 63%, MetALD 73%, ALD 89%) compared to those without SLD (43%) and cryptogenic SLD (40%; p<0.001). Anthropometric parameters differed substantially across groups, with all SLD subtypes showing higher BMI (27–29 kg/m²) and waist circumference (102–106 cm) compared to those without SLD (25 kg/m² and 91 cm, respectively; all p<0.001).

Laboratory values revealed expected patterns consistent with metabolic and alcohol-related hepatic dysfunction. Liver enzymes were progressively elevated across SLD groups, with the highest values observed in ALD participants: median AST (19, 22, 24, 27, 21 U/L), ALT (18, 26, 28, 31, 23 U/L), and GGT (21, 33, 43, 70, 28 U/L) for no SLD, MASLD, MetALD, ALD, and cryptogenic SLD, respectively (all p<0.001). Markers of glucose metabolism showed similar trends, with median fasting glucose (94, 102, 101, 103, 100 mg/dL) and HbA _1c_ (5.4%, 5.6%, 5.6%, 5.5%, 5.7%) varying across groups. HDL cholesterol was lower in all SLD groups (50–54 mg/dL) compared to those without SLD (61 mg/dL), while systemic inflammation markers including C-reactive protein were elevated (all p<0.001).

The prevalence of diabetes mellitus was substantially higher in all SLD groups compared to participants without SLD: MASLD 24%, MetALD 26%, ALD 20%, and cryptogenic SLD 28% versus 6% in those without SLD (p<0.001). Metabolic syndrome prevalence was similarly elevated, reaching 60% in MASLD, 57% in MetALD, 68% in ALD, and 65% in cryptogenic SLD compared to 24% in those without SLD (p<0.001).

Lifestyle factors differed significantly between groups. Alcohol consumption patterns varied by design, with ALD participants showing the highest prevalence of heavy drinking (≥20g pure alcohol/day: 89%), followed by cryptogenic SLD (79%) and MetALD (38%), compared to 7% in MASLD and 10% in no SLD (p<0.001). Ever-smoking rates were highest in ALD participants (67%), intermediate in MASLD and MetALD (53–54%), lowest in cryptogenic SLD (31%), and 46% in those without SLD (p<0.001). Proton pump inhibitor use was more common in SLD groups (8–10%) compared to those without SLD (6%; p<0.001). Hiatal hernia prevalence was elevated in ALD (65%), intermediate in MASLD (56%), MetALD (55%), and cryptogenic SLD (54%), compared to 51% in those without SLD (p=0.007).

**Fig. 1:**
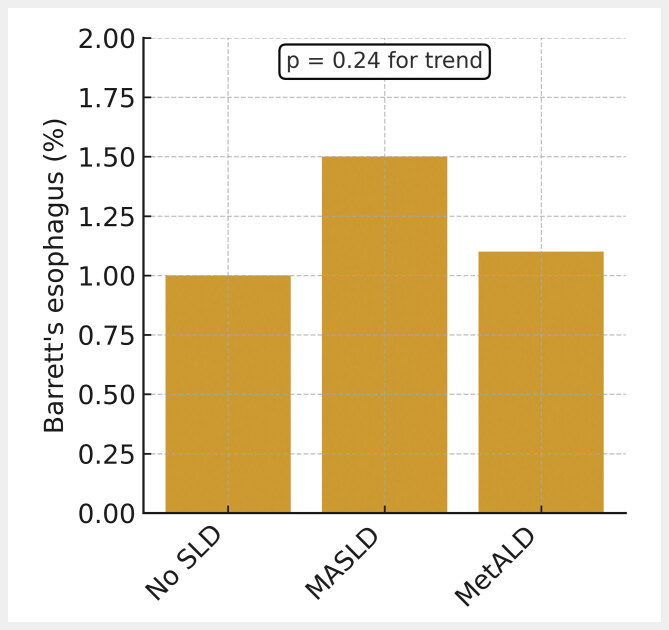
Percentage of Barrettʼs esophagus across the groups: individuals with no specific liver disease (No SLD), those with Metabolic Dysfunction-Associated Steatotic Liver Disease, and those with Metabolic Alcohol-Related Liver Disease. The data show no significant difference in the prevalence of Barrettʼs esophagus between the groups (p=0.28), indicating that the presence of this condition does not vary significantly across different liver disease types in this study.

BE was diagnosed in 62 participants (1.1% overall), with prevalence varying non-significantly across SLD groups: 29 cases (1.0%) in participants without SLD, 30 cases (1.5%) in MASLD, 2 cases (0.9%) in MetALD, 1 case (1.3%) in ALD, and no cases in cryptogenic SLD (p=0.24) ( Fig. 1 ). The majority of cases were non-dysplastic BE (n=59, 95%), with only 2 cases of low-grade dysplasia and 1 case of high-grade dysplasia identified.

### Association Between SLD and BE

In unadjusted logistic regression analysis using no SLD as the reference category, none of the SLD subtypes showed a significant association with BE ( Table 2 ). MASLD was associated with an odds ratio of 1.52 (95% CI: 0.91–2.53, p=0.112), MetALD with 0.91 (95% CI: 0.22–3.85, p=0.902), and ALD with 1.29 (95% CI: 0.17–9.62, p=0.801).

**Table:** **Table 2** Association between SLD subtypes and the presence of BE. Values are reported as odds ratios with 95% confidence intervals, derived from multivariable logistic regression models adjusted for demographic, metabolic, and clinical covariates.

SLD Type	Barrettʼs Cases n/N (%)	Model 1 Unadjusted	Model 2 Age + Sex Adjusted	Model 3 Fully Adjusted†
**No SLD**	29/2957 (1.0)	1.00 (Reference)	1.00 (Reference)	1.00 (Reference)
**MASLD**	30/2027 (1.5)	1.52 (0.91–2.53) p = 0.112	1.17 (0.69–1.97) p = 0.558	1.28 (0.74–2.21) p = 0.386
**MetALD**	2/223 (0.9)	0.91 (0.22–3.85) p = 0.902	0.61 (0.14–2.58) p = 0.501	0.66 (0.16–2.85) p = 0.582
**ALD**	1/79 (1.3)	1.29 (0.17–9.62) p = 0.801	0.76 (0.10–5.73) p = 0.794	0.80 (0.11–6.07) p = 0.830
**Cryptogenic SLD**	0/221 (0.0)	–	–	–

After adjustment for age and sex (Model 2), the associations between SLD subtypes and BE remained non-significant and were further attenuated. MASLD showed an adjusted odds ratio of 1.17 (95% CI: 0.69–1.97, p=0.558), MetALD 0.61 (95% CI: 0.14–2.58, p=0.501), and ALD 0.76 (95% CI: 0.10–5.73, p=0.794). In this model, female sex was strongly protective against BE (OR 0.23, 95% CI: 0.12–0.45, p<0.001), while age showed a non-significant trend toward increased risk (OR 1.02 per year, 95% CI: 0.99–1.05, p=0.122).

In the fully adjusted model (Model 3) including age, sex, metabolic syndrome, proton pump inhibitor use, hiatal hernia, and smoking status, no SLD subtype demonstrated a significant association with BE. MASLD showed an odds ratio of 1.28 (95% CI: 0.74–2.21, p=0.386), MetALD 0.66 (95% CI: 0.16–2.85, p=0.582), and ALD 0.80 (95% CI: 0.11–6.07, p=0.830). The strongest independent predictor of BE in this model was hiatal hernia (OR 2.05, 95% CI: 1.16–3.60, p=0.013), followed by the protective effect of female sex (OR 0.26, 95% CI: 0.13–0.50, p<0.001). Ever-smoking showed a non-significant positive association (OR 1.52, 95% CI: 0.90–2.58, p=0.119).

### Interaction Analyses

Formal testing for interactions identified two statistically significant effects. First, the relationship between MASLD and BE varied by age (p=0.042), with the association becoming weaker as age increased (interaction OR 0.95 per year, 95% CI: 0.90–1.00). Second, a significant interaction was observed between MASLD and smoking status (p=0.025), suggesting a stronger association with BE among ever-smokers (interaction OR 3.64, 95% CI: 1.18–11.27).

No significant interactions were detected between SLD subtypes and sex, metabolic syndrome, proton pump inhibitor use, or hiatal hernia (all p>0.05). However, several analyses had limited statistical power due to small subgroup sizes and zero events in certain strata. This was particularly problematic for interactions involving MetALD, ALD, and cryptogenic SLD with female sex, where perfect prediction occurred due to absence of Barrettʼs cases in specific subgroups.

### Secondary Analysis: Fibrosis/Steatosis Markers in Participants Without SLD

Among participants without clinically defined SLD, none of the non-invasive liver fibrosis markers showed a significant association with BE risk. The FIB-4 index was associated with an odds ratio of 1.06 (95% CI: 0.83–1.36, p=0.649), while the APRI score showed an odds ratio of 1.17 (95% CI: 0.73–1.90, p=0.516). The fatty liver index showed a borderline non-significant association (OR 1.007 per unit increase, 95% CI: 0.998–1.016, p=0.108), potentially indicating a weak relationship between subclinical hepatic steatosis and BE risk.

Among participants with available elastography data, neither controlled attenuation parameter (CAP) values (OR 1.004 per unit, 95% CI: 0.996–1.012, p=0.324) nor liver stiffness measurements (OR 0.98 per kPa, 95% CI: 0.79–1.22, p=0.853) were associated with BE prevalence.

## Discussion

### Principal Findings

In this large, population-based screening study of over 5000 symptom-free adults, we found no evidence for a strong independent association between any SLD and BE. The prevalence of BE was consistently low (around 1%) and did not change after adjusting for age, sex, metabolic syndrome, proton pump inhibitor use, smoking, and hiatal hernia. In the fully adjusted model, the only independent predictors were hiatal hernia and male sex, with women having about one-third the risk compared to men. Because both sexes were invited from age 45 onwards and age was included as a covariate in all multivariable models, the lower risk observed in females is unlikely to be explained by sex-specific differences in screening age.

### Comparison with Previous Work

Published data on a potential liver–esophagus axis remain limited and inconsistent. Some small case–control series suggest a positive association between hepatic steatosis and BE, whereas other observational studies report null findings; robust population-based evidence is still lacking 23 24 25 26 .

All were limited by heterogeneous steatosis definitions, inadequate control for reflux‑related factors, and insufficient power. Our study addresses these limitations by applying contemporary diagnostic criteria for SLD, incorporating systematic upper endoscopy with histologic confirmation, and adjusting for the full spectrum of established Barrett’s risk factors in a sample size an order of magnitude larger than prior series.

### Pathophysiological Considerations

These largely null findings may be biologically plausible. Barrett’s metaplasia is driven predominantly by chronic mucosal injury from acid and bile reflux, mechanical stress related to hiatus hernia, and local inflammatory cascades 27 28 29 .

Although MASLD is a marker of systemic low‑grade inflammation and insulin resistance, these circulating alterations may be insufficient to overcome the dominant effect of local reflux‑mediated damage 30 31 32 .

The significant interaction between MASLD and age, suggesting a weaker association in older individuals, is hypothesis‑generating but should be interpreted cautiously given the wide confidence intervals and lack of a dose–response gradient.

### Strengths and Limitations

This study was based on a fixed screening cohort, and formal power calculations were therefore performed post hoc. Among the 5507 included participants, the prevalence of BE was approximately 1.1–1.2%, and the proportion with MASLD and MetALD was 39% and 5%, respectively. Assuming a two-sided α of 0.05, the observed outcome prevalence provided adequate (>80%) power to detect clinically relevant associations with odds ratios of about 1.8 or greater for the comparison of MASLD versus no SLD, but limited power for smaller effect sizes and for rarer SLD phenotypes such as MetALD and ALD. Consequently, while our findings argue against a strong independent association between SLD and BE, more modest risk elevations—particularly in smaller subgroups and interaction analyses – cannot be definitively excluded. Key strengths of the study include the prospective design, comprehensive phenotyping, use of current MASLD/MetALD definitions, and prespecified multivariable modeling with interaction testing. Misclassification bias was minimized by requiring both endoscopic and histologic criteria for Barrett’s diagnosis. We did not systematically collect detailed data on reflux symptoms or family history of esophageal or gastric cancer, which may contribute to residual confounding and limits our ability to fully adjust for these established Barrett’s risk factors.

The principal limitation is statistical power for very small effect sizes; with a Barrett’s prevalence of 1.2 % our study could reliably detect odds ratios ≥1.8. Cross‑sectional design precludes causal inference, and the single‑center European setting may limit generalizability to populations with higher Barrett’s rates. Another key limitation of our study is the potential for healthy-volunteer and selection bias. Participation in the Sakkopi screening program and consent to concomitant upper endoscopy were voluntary, and such combined examinations are not standard practice in most European countries. Thus, our cohort likely overrepresents health-conscious individuals willing to undergo extensive screening, which may limit the generalizability of our findings to the broader population and to patients undergoing endoscopy for clinical indications. SLD was defined by abdominal ultrasonography, which is less sensitive than VCTE/CAP for detecting mild steatosis and cannot reliably quantify liver fat or fibrosis, leaving room for misclassification. Nevertheless, ultrasound is currently the most practical first-line modality in population-based endoscopy programs due to its availability, non-invasiveness, and low cost. While integration of VCTE or elastography with CAP into colorectal cancer screening pathways could improve phenotyping of hepatic steatosis and fibrosis, this would require additional equipment, trained personnel, and examination time, and is not yet routinely implemented in most screening settings. Our findings should therefore be interpreted in light of this methodological constraint.

### Clinical and Research Implications

Our results do not support incorporating metabolic SLD status into algorithms for BE screening or surveillance. Endoscopic resources should remain concentrated on well-established high-risk groups, namely older men with chronic reflux symptoms and individuals with hiatal hernia. Future studies should investigate whether subclinical steatosis – detected through imaging or biomarkers in younger populations – interacts with early-life reflux exposure, and prospective research is needed to assess incident BE in patients with progressive liver fibrosis. In the interim, performing combined screening for varices and Barrett’s during portal hypertension surveillance may be justified for logistical efficiency, but not due to any intrinsic increase in Barrett’s risk.

## Conclusions

In this large, population-based cohort, SLD was not independently associated with BE after rigorous adjustment for demographic, metabolic, and gastroesophageal risk factors. The null association suggests that SLD is unlikely to play a major role in Barrett’s pathogenesis, which appears to be driven predominantly by reflux-related mechanisms such as hiatal hernia and lower esophageal sphincter dysfunction. These results have direct implications for precision prevention, reinforcing the need to concentrate screening and surveillance strategies on established, high-yield risk factors while avoiding the unnecessary expansion of screening criteria to low-risk populations.

## Statements

### Author Contribution

All authors contributed to the development of the manuscript and approved its final version.

### Data Sharing Statement

The data associated with this submission can be made available upon a reasonable request.

### Ethics Statement

The study and all procedures were conducted in accordance with the principles outlined in the Declaration of Helsinki. Approval for the study protocol was obtained from the local ethics committee for the province of Salzburg, with the assigned approval number 415-E/1262. Written informed consent was obtained from each participant involved in the study.
